# An autophagy-related four-lncRNA signature helps to predict progression-free survival of neuroblastoma patients

**DOI:** 10.3389/fonc.2022.1014845

**Published:** 2022-12-01

**Authors:** Jing Wang, Xinyao Meng, Ke Chen, Jiexiong Feng

**Affiliations:** Department of Pediatric Surgery, Tongji Hospital, Tongji Medical College, Huazhong University of Science and Technology, Wuhan, China

**Keywords:** autophagy, neuroblastoma, progression-free survival, prognosis, long non-coding RNA

## Abstract

**Background:**

This study aimed to identify autophagy-related long non-coding RNAs (lncRNAs) associated with progression of neuroblastoma (NB), and to build an autophagy-related lncRNA signature that helps to predict progression-free survival (PFS) of NB.

**Methods:**

Three independent gene expression datasets were utilized in this study. Autophagy-related genes (ARG) associated with PFS of NB patients were firstly identified by univariate Cox survival analysis. lncRNAs correlated with those PFS-related ARGs were then identified. The least absolute shrinkage and selection operator (LASSO) regression and multivariate Cox regression analyses were performed to select out those lncRNAs with the best prognostic value for PFS. The Receiver Operating Characteristic (ROC) and Area Under Curve (AUC) analyses were performed to assess the prediction accuracy.

**Results:**

Four autophagy-related lncRNAs (AL356599.1, AC022075.1, AC020928.1 and LINC02076) were found to be with the best prognostic value and integrated into a four-lncRNA risk signature for predicting PFS of NB patients. The four-lncRNA signature significantly stratify NB patients into two risk groups, with high-risk group has significantly poorer PFS than the low-risk group. The prognostic role of the lncRNA signature was independent with other clinical risk factors. The ROC curves revealed that the lncRNA signature has a good performance in predicting PFS (AUC > 0.70). A nomogram based on COG (Children’s Oncology Group) risk and the lncRNA risk score was constructed, showing good prediction accuracy (C-index = 0.700). The prognostic ability of the nomogram was better than that of COG risk alone (AUC = 0.790 versus AUC = 0.748). GSEA analyses revealed that multiple autophagy-related gene sets are significantly enriched in the low-risk group.

**Conclusions:**

We identified an autophagy-related four-lncRNA signature that could help to predict the PFS of NB patients. Autophagy-related gene sets are significantly enriched in low-risk group, suggesting tumor suppressive roles of autophagy in NB.

## Introduction

Neuroblastoma (NB) is the most common extracranial solid tumor of childhood, originating from the adrenal medulla or paraspinal regions where sympathetic nervous tissue is present. It affects about 1 in 7000 live births, and about 650 cases per year are diagnosed in the United States (1, 2). Nowadays, most patients diagnosed with NB in North America are treated according to the Children’s Oncology Group (COG) risk stratification system, and the COG is also in the process of revising the COG risk stratification schema. Based on age at diagnosis, MYCN amplification status, International Neuroblastoma Staging System (INSS) stage, histopathology and tumor cell ploidy, NB patients are stratified into low-, intermediate-, and high-risk groups according to the 2007 COG risk system (2, 3). The latest available data reveals that the 5-year overall survival (OS) rate was about 97% for the low-risk group (4); the 3-year OS rate was about 96% for the intermediate-risk group (5); while the overall survival rate for patients with high-risk NB is only about 50% despite multimodal aggressive therapy (3). Recurrence of the original NB tumor remains a major contributor of mortality, accounting for about 67% of total deaths (6). Patients with high-risk NB who were aggressively treated may even develop late recurrences more than 5 years after completion of therapy (6, 7). Thus, further improvement of the risk stratification system for predicting progression-free survival (PFS) may help to the management of NB patients.

The importance of autophagy in the development of malignancies has gained increasing attention since the Nobel Prize for Physiology or Medicine was awarded to Yoshinori Ohsumi for his work on the mechanism of autophagy in 2016 (8–10). Autophagy plays context-dependent roles in cancers, either can be tumor-suppressive or can be tumor-protective (10). Recently, strategies that stimulate or inhibit autophagy have also been suggested as cancer therapies (10–12). Studies have also been focusing on developing novel biomarkers that can be used to monitor autophagy and thus help to guide autophagy-related therapeutic strategies for cancer patients (10). The implications of autophagy in NB have also been reported in recent years (13–18), however, the association between autophagy and the progression of NB is still largely unknown.

In this study, we performed integrated analyses of transcriptome profiles of NB tissues by combing one RNA-seq datasets (TARGET NBL, n= 153) and two microarray datasets (GSE49710 and E-MTAB-8248, n = 498 and n =223 respectively) in order to get a comprehensive understanding of the relationship between autophagy and progression of NB. We focus on identifying autophagy-related lncRNAs that could help to predict PFS of NB patients in this study.

Finally, four autophagy-related lncRNAs were found to be with the best prognostic values and were integrated into a four-lncRNA risk signature for predicting PFS of NB patients. The four-lncRNA signature performs well in predicting PFS of NB patients and also improves the PFS prediction ability of COG risk classification. Gene Set Enrichment Analysis (GSEA) revealed that multiple autophagy-related gene sets were significantly enriched in the low-risk group, while no autophagy gene set was enriched in the high-risk group, indicating that autophagy tend to play tumor-suppressive roles in NB.

## Materials and methods

### Neuroblastoma datasets processing

The RNA-sequence transcriptome expression profiles of NB tissues (TARGET NBL, n = 153) were obtained from the National Cancer Institute GDC Data Portal. One of the transcriptome expression microarray profiles (GSE49710, n = 498) was obtained from Gene Expression Omnibus (GEO) database, the other one (E-MTAB-8248, n = 223) was obtained from ArrayExpress database. The clinical characteristics of the three cohorts are shown in **Additional file 1 (Table S1)**. The RNA-sequence dataset (TARGET NBL) was used for initial exploration and termed as cohort 1. The microarray datasets (GSE49710 and E-MTAB-8248) were used for validation and termed as cohort 2 and cohort 3 respectively. Both of GSE49710 and E-MTAB-8248 are Aiglent microarrays performed on platform GPL16876 (Agilent-020382 Human Custom Microarray 44k). The Agilent microarray probes IDs were firstly annotated using the platform GPL16876; Then, the probes IDs were re-annotated according to their corresponding Genebank Accession number in order to renew the annotation. Finally, the Ensemble ID in the three datasets (TARGET NBL, GSE49710 and E-MTAB-8248) were transformed into gene symbols according to GRCh38.p12 in order to keep consistent with each other. The background of the three datasets were intersected adjusted. When multiple probes mapped to a same gene, the mean of the signal intensities will be used.

### Construction of the autophagy-related lncRNA prognostic signature

Autophagy-related genes (ARGs) were obtained from Human Autophagy Database (HADb) (https://www.autophagy.lu/), with a total of 232 ARGs. Univariate Cox regression analyses were utilized to identify those ARGs associated with PFS of NB patients in cohort 1. A p-value of ≤ 0.5 was considered statistically significant. Any events (including death, relapse, metastasis, or progression) occurred during follow-up was considered as progression. The LncRNA of which the expression level is significantly correlated with the expression level of those ARGs with Pearson’s correlation coefficient (r) ≥ 0.5 will be extracted as autophagy-related lncRNAs. Only those lncRNAs matched to GENCODE annotation of long non-coding RNA (release 31, GRCh38.p12) were selected. Those PFS-related lncRNAs were put into the least absolute shrinkage and selection operator (LASSO) penalty Cox regression model and multivariate Cox regression model survival analysis to eliminate false positives due to over-fitting (19). Finally, the autophagy-related lncRNA prognostic signature was constructed by weighting the Cox regression coefficients to calculate a risk score for each patient. The median value of the risk score was chosen as the cut-off value and the patients were group into low-risk and high-risk group accordingly. The same formula and the same cut-off value were applied to the validation cohorts.

### Cell culture

Human neuroblastoma cell lines (BE2C, IMR-32, SH-SY5Y and SK-N-AS) and human embryonic kidney 293 (HEK293) cells were used for further researches. Cell lines BE2C and IMR-32 were cultured in MEM contained with 10% fetal bovine serum, and HEK293 and SK-N-AS in DMEM with 10% fetal bovine serum, and cell line SH-SY5Y in DMEM/F-12 contained with 10% fetal bovine serum. The cells were cultured at 37°C in an incubator with 5% CO_2_ and passaged by 0.1% trypsin digestion every 3–4 days during the logarithmic growth period. All cells were grown addictively. To detect the association of lncRNAs and autophagy, cells were applied with 100 nM Rapamycin for 48h for further investigation.

### Quantitative real-time polymerase chain reaction and western blot

Total RNA was obtained from tissues using TRIzol reagent as described by the manufacturer (Invitrogen Life Technologies Co, USA). Quantification of extracted RNA was performed using NanoDrop. For the mRNA detection, Real-time PCR was performed by using an SYBR Premix Ex Taq kit (TOYOBO Biotechnology, Japan) on a StepOnePlus Real-time PCR System (Applied Biosystems, Foster City, CA, USA), along with the Glyceraldehyde 3-phosphate dehydrogenase (GAPDH) as the endogenous control. The Ct value was calculated based on the ΔΔCt method. Fold change of gene expression was expressed as 2^-ΔΔCt^. The primers used in this study were as follows: AL356599.1, sense strand 5′- GGGTCAGTCAACAAGGTCAGTCAAG-3′, antisense strand 5′- AACACCGCTCATCCTGGCAATTAG-3′, AC022075.1, sense strand 5′-CCTTGCTCGACCTTTGGTGA-3′, antisense strand 5′-GGAGGTAAAACCCGACAGGG-3′, AC020928.1, sense strand 5′-CAAGGCCTCCACCTGATGAA-3′, antisense strand 5′-CGTCTACGCCATTGTAGGGG-3′, and LINC02076, sense strand 5′- GGAGGGTGGAAAAGAAGACGATGAG-3′, antisense strand 5′- CAGCATGTTGGTCAGGCAGGTC-3′Total protein was extracted using RIPA lysis buffer with PMSF and Cocktail added. Protein concentrations were determined by the BCA method. Protein bands were quantified by densitometry with Quantity One Software.

### Statistical analysis and plots construction

The univariate and multivariate Cox proportional hazards regression analyses were performed by the R package “survival”. The LASSO Cox survival analyses were performed by the R package “glmnet”. The Pearson correlation matrix was generated by the R package “corrplot”. The violin plots were constructed by the R package “ggpubr”, and the differences between groups were compared by t-test. The Kaplan–Meier survival plots were constructed by R software (package “survival” and “survminer”) or GraphPad Prism 5, and the statistical significance was assessed using the two-sided log-rank test. The receiver operating characteristic (ROC) curves and area under curve (AUC) analyses were performed by the R package “timeROC” and “survivalROC”. The alluvial diagrams were generated by the R package “ggalluvial”. The nomogram was constructed by R package “rms”. Gene Set Enrichment Analysis (GSEA) was performed by GSEA software (version 4.0.03), and a nominal p-value < 0.05 as well as false discovery rate (FDR) q-value < 0.25 were considered statistically significant. The combined multiple GSEA plot was constructed by R package “plyr”, “ggplot2”, “grid” and “gridExtra”. The R software version 4.0.0 was utilized in this study. All statistical tests were two-sided and p-value < 0.05 was considered statistically significant. Data were analyzed using the GraphPad Prism software package (version 5; GraphPad Software Inc., La Jolla, CA, USA) and are presented as the mean ± standard error of the mean. Differences between two groups were analyzed using an unpaired t-test with Welch’s correction. Analysis of variance (ANOVA) was used to compare data of more than two groups.

## Results

### Identification of autophagy-related lncRNAs associated with progression-free survival

**Figure 1:**
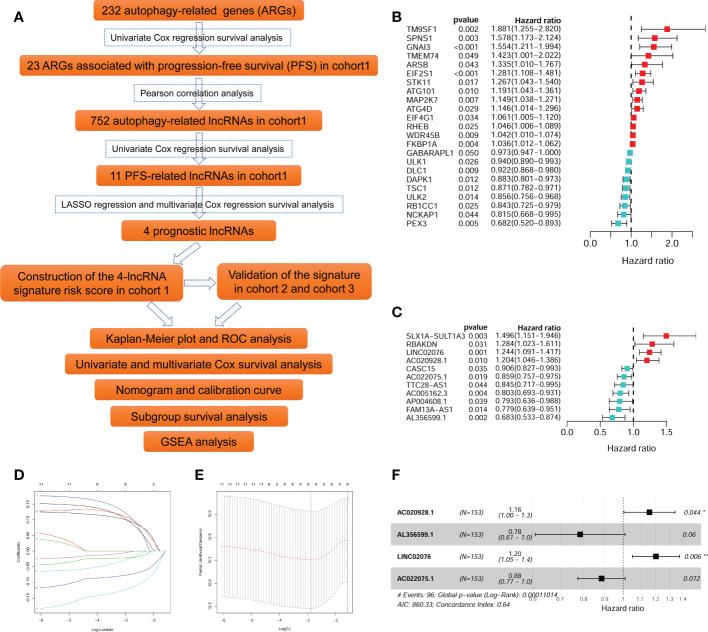
Identification of autophagy-related lncRNAs associated with PFS. **(A)** The flowchart of this study. **(B)** The univariate survival analysis of the 23 ARGs associated with PFS of NB patients in cohort 1. **(C)** The univariate survival analysis of the 11 autophagy-related lncRNAs associated with PFS of NB patients in cohort 1. **(D, E)** The LASSO regression analysis. **(F)** The multivariate survival analysis of the four lncRNAs with the best prognostic value for PFS. ARG, autophagy-related gene; PFS, progression-free survival; lncRNA, long noncoding RNA; NB, neuroblastoma; LASSO, least absolute shrinkage and selection operator. *p < 0.05 and **p < 0.01.

The flow diagram recapitulating this study is showing in **Figure 1A**. Firstly, univariate Cox survival analyses were performed for the 232 ARGs in cohort 1 (TARGERT NBL, n = 153). A total of 23 ARGs were identified to be significantly correlated with PFS of NB patients (**Figure 1B**). Pearson correlation analyses were then performed to identify those lncRNAs significantly correlated with each of those 23 PFS-related ARGs. A total of 752 autophagy-related lncRNA were identified, however, only 11 of them were significantly associated with PFS (**Figure 1C**). Then the 11 PFS-related lncRNAs were put into LASSO regression (**Figures 1D, E**) and nine of them were selected out. Finally, the multivariate Cox regression model were utilized and four autophagy-related lncRNAs (AL356599.1, AC022075.1, AC020928.1 and LINC02076) were screened out as being with the best prognostic value for PFS of NB patients. Two of them (AL356599.1 and AC022075.1) are protective factors for PFS of NB patients, while the other two (AC020928.1 and LINC02076) are risk factors (**Figure 1F**). The differential analysis of these four lncRNAs in Pan-cancer (**Additional File 2, Figure S2**). The expression of AL356599.1, AC022075.1, AC020928.1 and LINC02076 exhibited significant difference between normal and tumor tissue in most types of cancer.

**Figure 2:**
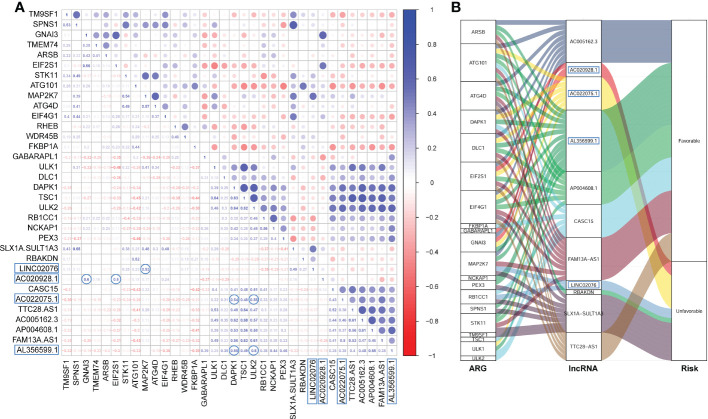
The Pearson correlation among the ARGs and lncRNAs. **(A)** The Pearson correlation matrix. **(B)** The alluvial plot represents the correlation between the ARGs and the lncRNAs (Pearson correlation threshold ≥ 0.5). ARG, autophagy-related gene; lncRNA, long noncoding RNA.

The Pearson correlation among these PFS-related ARGs and lncRNAs were shown in the correlation matrix (**Figure 2A**) and the alluvial diagram (**Figure 2B**). Both of AL356599.1 and AC022075.1 are significantly positively corelated (with r ≥ 0.5) with autophagy-related genes DAPK1 and ULK2, respectively. AC020928.1 is significantly positively correlated (with r ≥ 0.5) with autophagy-related genes GNAI3 and EIF2S1, respectively. LINC02076 is significantly positively correlated (with r ≥ 0.5) with autophagy-related gene MAP2K7.

### Clinical relevance of the four prognostic autophagy-related lncRNAs

**Figure 3:**
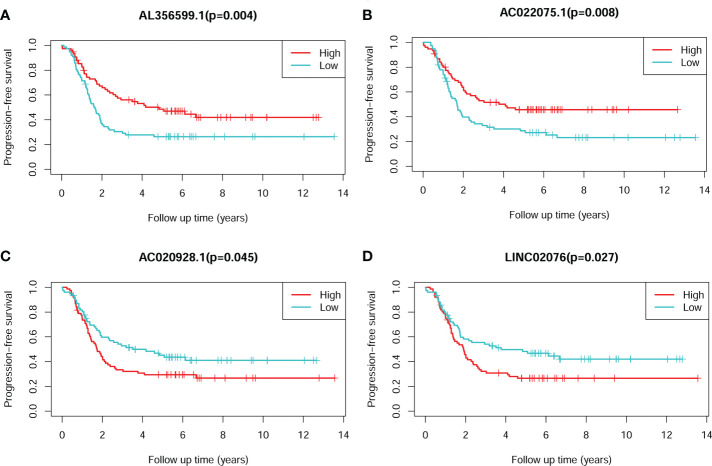
The Kaplan-Meier survival plots of the four lncRNAs for PFS of NB patients. **(A)** AL356599.1. **(B)** AC022075.1. **(C)** AC020928.1. **(D)** LINC02076. The Log-rank tests p-values were shown. PFS, progression-free survival; lncRNA, long noncoding RNA; NB, neuroblastoma.

The Kaplan-Meier plots showed that each of the four lncRNA could significantly stratify NB patients in cohort 1 into two risk groups, respectively, with the median expression values as the cut-of values (**Figure 3**). High expression of AL356599.1 and AC022075.1 are associated with relative good PFS (**Figures 3A, B**), while high expression of AC020928.1 and LINC02076 are associated with relative bad PFS (**Figures 3A, B**).

**Figure 4:**
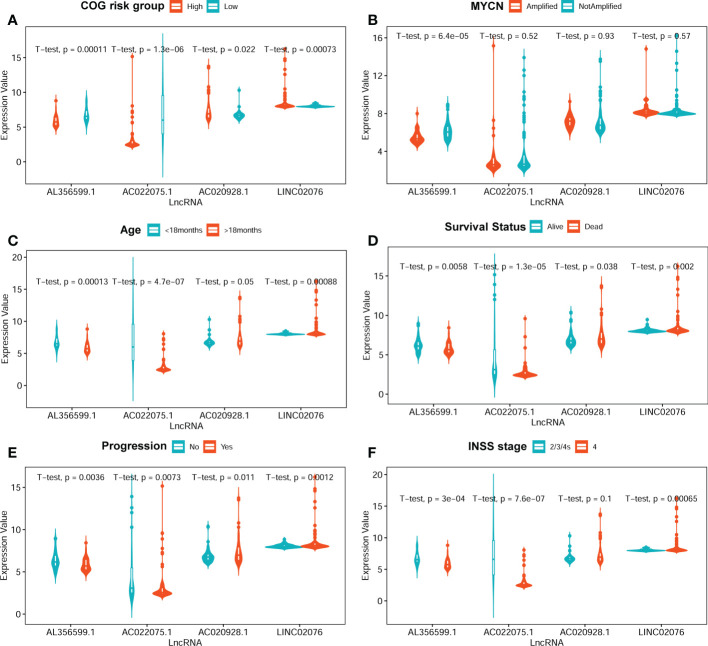
The average expression levels of the four lncRNA between different subgroups. **(A)** COG risk group (high vs low). **(B)** MYCN amplification (amplified vs not-amplified. **(C)** Age (< 18 months vs > 18 months). **(D)** Survival status (alive vs dead). **(E)** Progression (no vs yes). **(F)** INSS stage (2/3/4S vs 4). The two-sided t-tests p-values were shown. COG, Children’s Oncology Group; lncRNA, long noncoding RNA.

The average expression values of the four lncRNA between different clinical subgroups are showed in **Figure 4**. The comparisons were performed between subgroups based on COG risk group (high versus low, **Figure 4A**), MYCN amplification status (amplified versus not-amplified, **Figure 4B**), age (< 18months versus > 18mouths, **Figure 4C**), survival status (death versus alive, **Figure 4D**), progress (yes versus no, **Figure 4E**), and INSS stage (2/3/4S versus 4, **Figure 4F**). As we can see in **Figure 4**, the average expression levels of each of the four lncRNA were significantly different between low COG risk group and high COG risk group (**Figure 4A**), death group and alive group (**Figure 4D**), and progress group and non-progression group (**Figure 4E**). However, the expression levels of three lncRNAs (AC022075.1, AC020928.1 and LINC02076) between MYCN amplified and MYCN not-amplified groups are not significantly different, suggesting that the prognostic role of these three lncRNAs are independent with MYCN amplification status.

### Construction and validation of the four-lncRNA prognostic signature

**Figure 5:**
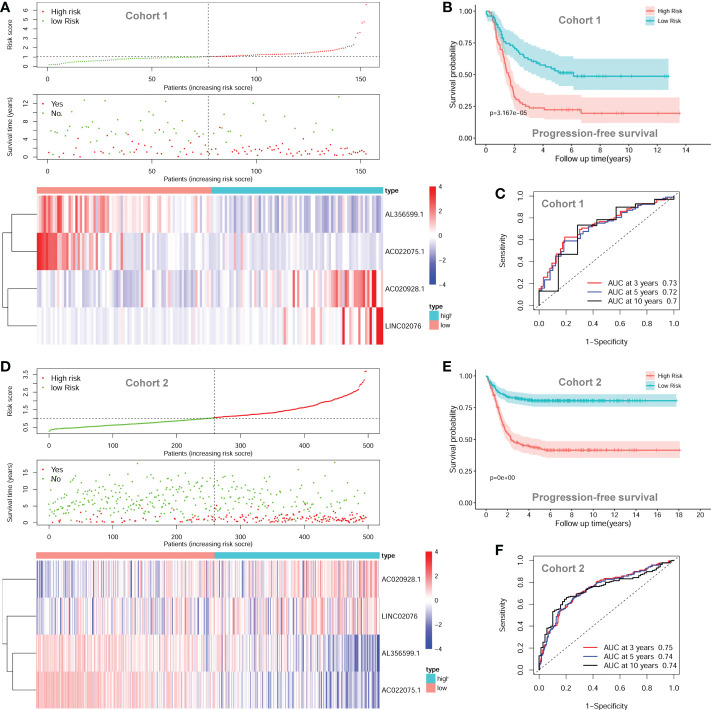
The autophagy-related four-lncRNA signature risk score. **(A)** The distribution of risk scores, survival status of each patient, and heatmap of lncRNAs expression pattern in cohort 1. **(B)** Kaplan-Meier survival curve for PFS of patients in the low-risk group and high-risk group for cohort 1. **(C)** Time-dependent ROC curves of the lncRNA signature in cohort 1. **(D)** The distribution of risk scores, survival status of each patient, and heatmap of lncRNAs expression pattern in cohort 2. **(E)** Kaplan-Meier survival curve for PFS of patients in the low-risk group and high-risk group for cohort 2. **(F)** Time-dependent ROC curves of the lncRNA signature in cohort 2. lncRNA, long noncoding RNA; PFS, progression-free survival; ROC, Receiver Operating Characteristic; AUC, Area Under Curve.

The four-lncRNA signature risk score were constructed based on the multivariate coefficients and expression values of each of the lncRNA for each patient as the following formula: risk score = 0.1849*LINC02076 + 0.1480*AC020928.1- 0.1230*AC022075.1 - 0.2437*AL356599.1. Then the entire cohort 1 was classified into two risk groups according to the median value of the risk score. The risk score of each patient, PFS status of each patient, and heatmap of the expression pattern of each lncRNA are shown in **Figure 5A**. Kaplan-Meier plots show that patients with high risk score have a significantly poorer PFS than those with low risk score (**Figure 5B**). The 3-years, 5-years and 10-years PFS rates for patients with high risk score were 25.00%, 22.29% and 19.51%, respectively; whereas, the 3-years, 5-years and 10-years PFS rates for patients with low risk score were 61.88%, 52.94% and 48.68%, respectively. Time-dependent ROC curves reveal that the AUC of the lncRNA signature in predicting PFS of NB patients in cohort 1 at 3-years, 5-years and 10-years were 0.73, 0.72 and 0.70, respectively (**Figure 5C**), indicating good prediction performance.

The prognostic significance of the lncRNA signature was then tested in cohort 2 (n = 498) and cohort 3 (n = 223) according to the same formula respectively. The risk score of each patient, PFS status of each patient, and heatmap of the expression pattern of each lncRNA are shown in **Figure 5D**. Kaplan-Meier plots show that patients with high risk score have a significantly poorer PFS than those with low risk score in cohort 2 (**Figure 5E**). The 3-years, 5-years and 10-years PFS rates for the patients with high risk score were 47.03%, 43.20% and 41.48%, respectively; whereas, the 3-years, 5-years and 10-years PFS rates for patients with low risk score were 82.31%, 80.45% and 80.45%, respectively. Time-dependent ROC curves reveal that the AUC of the lncRNA signature in predicting PFS of NB patients in cohort 2 at 3-years, 5-years and 10-years were 0.75, 0.74 and 0.74, respectively (**Figure 5F**), indicating good prediction performance. The validation of the lncRNA signature in cohort 3 shows similar results (**Additional File 2, Figure S1**).

### Survival analysis for the lncRNA signature in entire combined cohort

Since the expression backgrounds were intersected adjusted among the three datasets, and the same formula and the same cut-off value were utilized, we decided to combine the three datasets together as one entire cohort for further analyses in order to obtain more reliable results.

**Figure 6:**
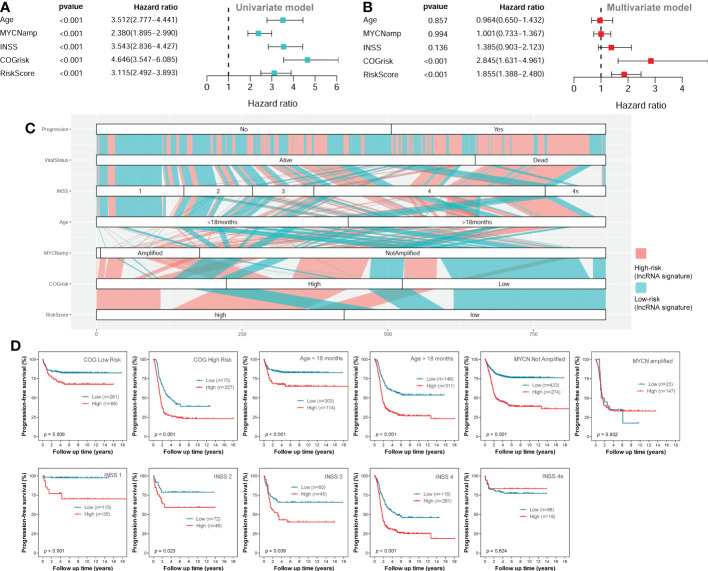
Survival analyses in the entire combined cohort. **(A)** The univariate survival analysis of the lncRNA signature and other clinical risk factors. **(B)** The multivariate survival analysis of the lncRNA signature and other clinical risk factors. **(C)** The alluvial diagram shows the relationship of lncRNA signature risk classification and other clinical risk factors. **(D)** The Kaplan-Meier plots show the PFS of different lncRNA signature risk classification (high-risk vs low-risk) in different subgroups of the entire combined cohort; the two-sided log-rank tests p-values were shown. lncRNA, long noncoding RNA; PFS, progression-free survival.

The univariate Cox regression survival analyses for the lncRNA signature risk score and other clinical risk factors in the entire combined cohort are shown in **Figure 6A**. The multivariate Cox regression survival analyses including the lncRNA signature risk sore and other clinical risk factors as covariates in the combined cohort are shown in **Figure 6B**. The risk factors are classified as follows: age (< 18 months vs ≥ 18 months), MYCN amplification (non-amplified vs amplified), INSS stage (INSS 1/2/3/4S vs INSS 4), COG risk (low vs high), and lncRNA risk score (low vs high). Since there are only several cases classified as COG intermediate-risk and the survival rate between COG intermediate-risk group and COG low-risk group are similar, we combined COG intermediate-risk group and COG low-risk group together as one COG low-risk group during the analysis. In the multivariate model, only the COG risk (HR = 2.845; 95%CI: 1.631-4.961; *p* < 0.001) and lncRNA signature risk score (HR = 1.855; 95%CI: 1.388-2.480, *p* < 0.001) were independently associated with PFS (**Figure 5B**).

As we can see in the alluvial plot of **Figure 6C**, a portion of patients in COG low-risk group are classified as lncRNA signature high-risk, while a portion of patients in COG high-risk group are classified as lncRNA signature low-risk. In the subgroup survival analysis for the entire cohort (**Figure 6D**), we can see the lncRNA signature could successfully stratify patients in the COG high-risk group or COG low-risk group into two risk groups for PFS. These results suggested that the four-lncRNA signature could help COG risk group to predict PFS of NB patients. The four-lncRNA signature also successfully stratified patients in different age groups into two risk groups for PFS (**Figure 6D**). Except for INSS 4S subgroup, the four-lncRNA signature successfully stratified each of the other INSS subgroups (1, 2, 3 and 4) into two risk group. However, while the four-lncRNA signature successfully stratified patients in MYCN not-amplified group into two risk groups, it fails to stratify patients in MYCN amplified group into two risk groups.

### Nomogram for prediction of progression-free survival

**Figure 7:**
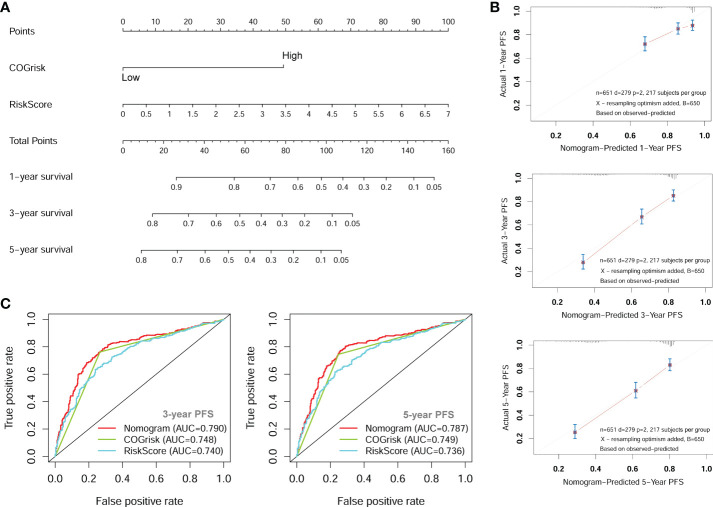
The nomogram for predicting of PFS of NB patients. **(A)** The nomogram incorporating COG risk classification and four-lncRNA risk score. **(B)** The calibration curves of the nomogram showing good consistency between predicted-PFS and actual-PFS. **(C)** The ROC curves showing the AUC of the COG risk classification and the AUC of the nomogram. lncRNA, long noncoding RNA; PFS, progression-free survival; COG, Children’s Oncology Group; ROC, Receiver Operating Characteristic; AUC, Area Under Curve.

Since the COG risk group classification already considered age, MYCN amplification and INSS stage into its risk classification system, we built a nomogram incorporating only the COG risk classification and the four-lncRNA signature risk score for prediction of PFS based on the entire combined cohort (**Figure 7A**). The C-index for the nomogram was 0.70. The 1-year, 3-year, and 5-year calibrate curves for nomogram all reveled that the predicted PFS was very close to the actual PFS (**Figure 7B**). The ROC curves analyses were also performed to compared the prognostic ability of the COG risk classification, the lncRNA signature risk and the nomogram (**Figure 7C**). We can see that the prediction ability of the nomogram was better than the COG risk alone (3-year AUC= 0.790 versus 3-year AUC=0.748). These results suggested that the four-lncRNA signature could improve the PFS prediction ability of COG risk classification system.

### GSEA analyses for different risk groups of the lncRNA signature

**Figure 8:**
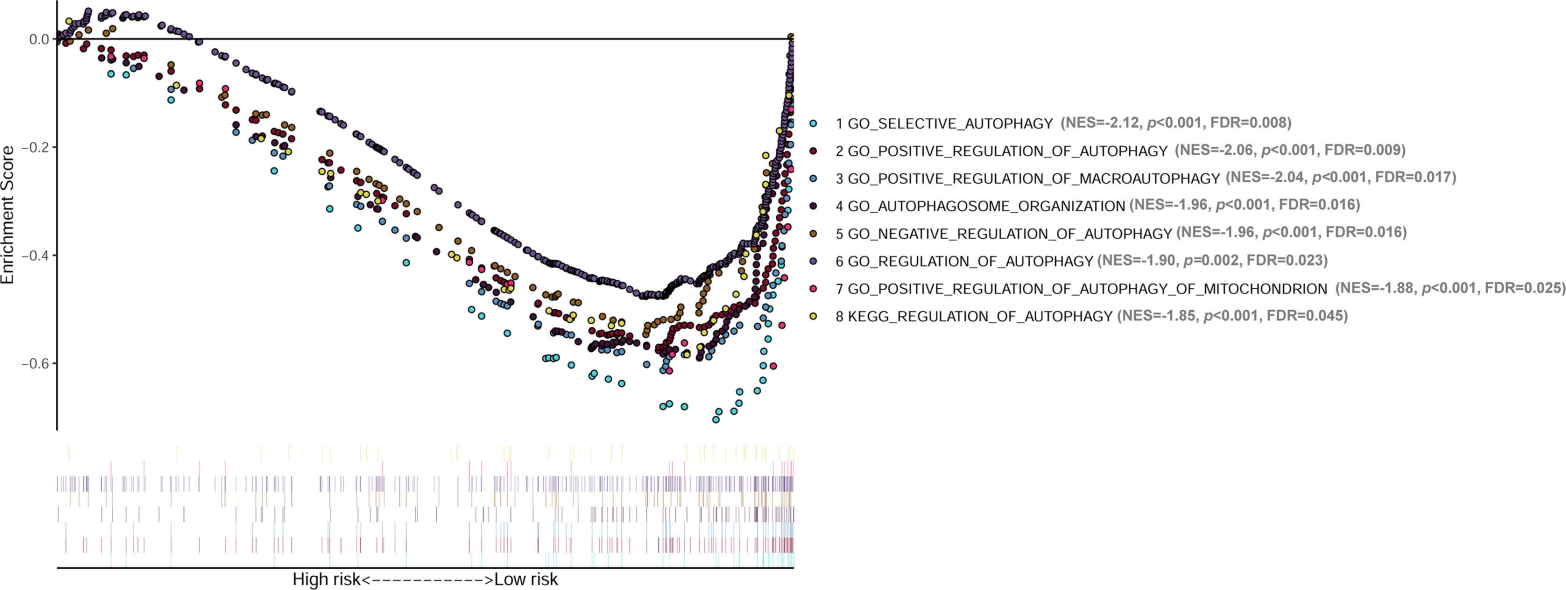
GSEA analyses showing the autophagy-related gene sets significantly enriched in the low-risk group of the lncRNA signature. No autophagy-related gene set is significantly enriched in the high-risk group. GSEA, Gene Set Enrichment Analysis; lncRNA, long noncoding RNA.

GSEA analyses were conducted to see the different gene sets enrichment between low-risk group and high-risk group generated by the lncRNA signature in cohort 1. The results revealed that no autophagy related gene set was enriched in the high-risk group, while multiple autophagy-related gene sets were significantly enriched in the low-risk group (**Figure 8**). Those gene-sets significantly enriched in the low-risk group includes GO selective autophagy, GO positive regulation of autophagy, GO positive regulation of macroautophagy, GO autophagosome organization, GO negative regulation of autophagy, GO regulation of autophagy, GO positive regulation of autophagy of mitochondrion, and KEGG regulation of autophagy. These results suggested that autophagy biological processes tend to play tumor suppressive roles in NB.

### Validation *in vitro*

**Figure 9:**
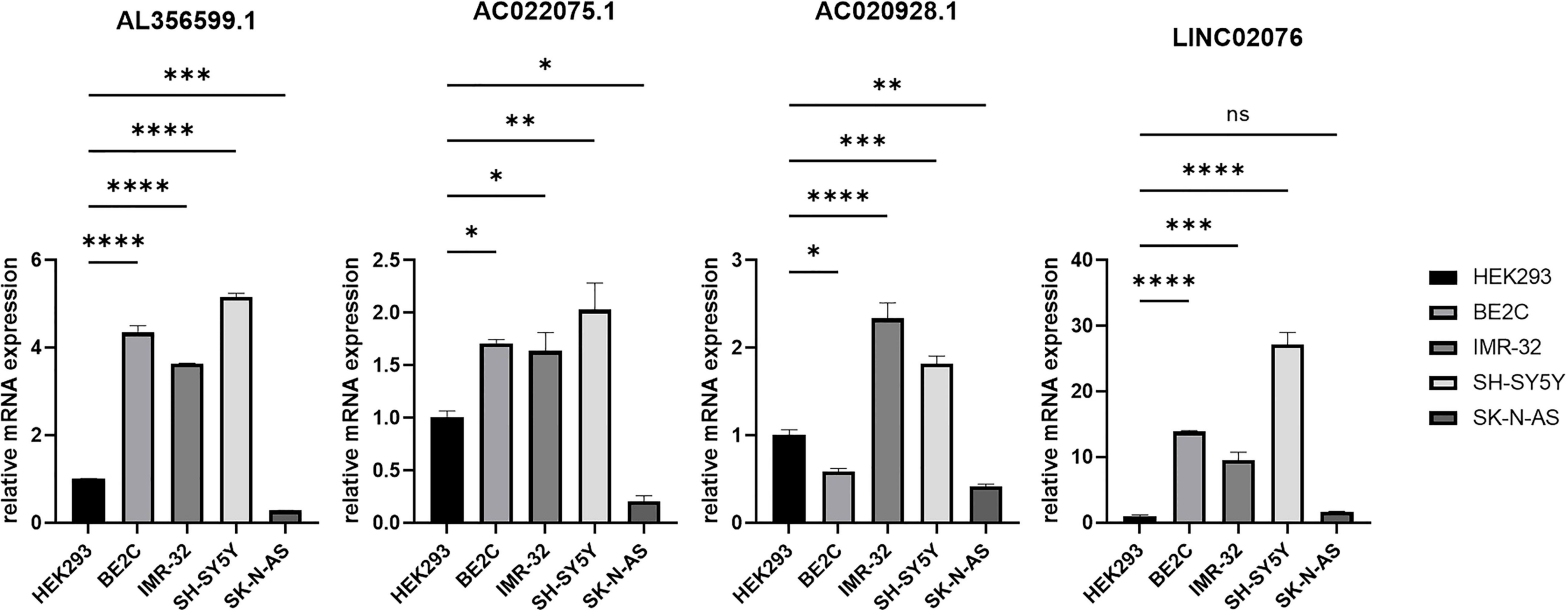
The expression of autophagy-related four-lncRNA in wild type cell lines. Results are representative of three independent experiments, ^ns^ *p* > 0.05; * *p* < 0.05; ** *p* < 0.01; *** *p* < 0.001; **** *p* < 0.0001.

**Figure 10:**
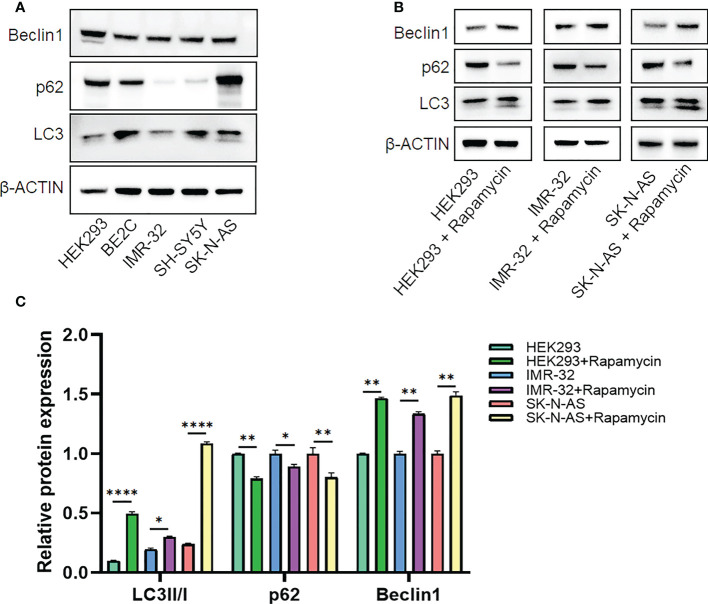
The expression of autophagy-related proteins. **(A)** Wild type cell lines. **(B, C)** Autophagy-activated cell lines. Results are representative of three independent experiments, * *p* < 0.05; ** *p* < 0.01; **** *p* < 0.0001.

**Figure 11:**
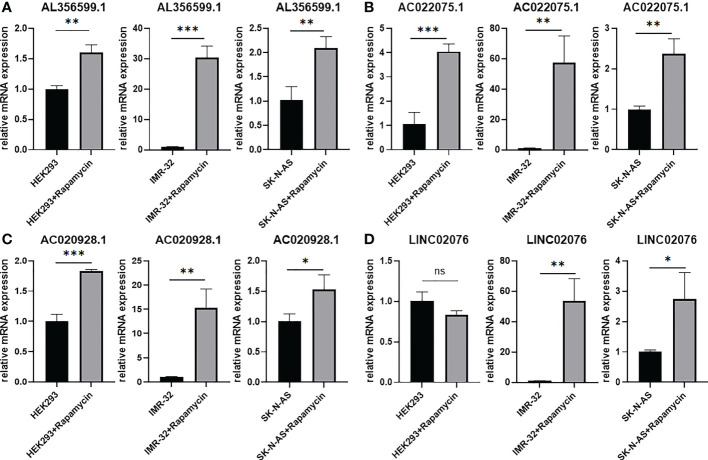
The expression of autophagy-related four-lncRNA **(A)** AL356599.1 **(B)** AC022075.1 **(C)** AC020928.1 **(D)** LINC02076 in autophagy-activated cell lines. Results are representative of three independent experiments, ^ns^ *p* > 0.05; * *p* < 0.05; ** *p* < 0.01; *** *p* < 0.001.

Candidate lncRNAs were verified in normal cell line HEK293 and human neuroblastoma cell lines BE2C, IMR-32, SH-SY5Yand SK-N-AS. As shown in **Figure 9**, the expression of the four lncRNAs selected was detected using qRT-PCR. AL356599.1, AC022075.1, AC020928.1 and LINC02076 were higher expressed in cell lines BE2C, IMR-32 and SH-SY5Y compared to HEK293, whereas in SK-N-AS were lower except LINC02076. To further explore the expression of these lncRNA in NB cell lines, we download the mRNA expression matrix of sixteen types of NB cell lines from the CCLE dataset (https://portals.broadinstitute.org/ccle). The expression of these lncRNAs in 16 types of NB cell lines were showed in **Additional file 2, Figure S3**, and the results showed that there is expression heterogeneity among these NB cell lines. Basically, the expression of these lncRNA in most of NB cell lines was higher than SK-N-AS. Furthermore, to detect the relation of these lncRNAs with autophagy, we stimulated the cells with Rapamycin to activate the autophagy and inspected autophagy-related proteins using western blot. As shown in the results, according to the expression of autophagy-related proteins in different wild type cell lines (**Figure 10A**), we selected IMR-32 and SK-N-AS cell lines stimulated by Rapamycin, and significant upregulation of LC3B and Beclin1 protein expression was observed in HEK293, IMR-32 and SK-N-AS, which suggested that autophagy was activated (**Figures 10B, C**). In addition, compared with normal cultured cells, the expression of AL356599.1, AC022075.1, AC020928.1 and LINC02076 was significantly increased in both IMR-32 and SK-N-AS cells after the addition of mTOR inhibitor (**Figure 11**), which suggested that these four lncRNAs were related to autophagy.

## Discussion

Autophagy is an intracellular homeostatic mechanism that delivers intracellular material into degradation and recycling, providing energy and molecular precursors for the cell itself (8–10). Autophagy has context-dependent roles in cancers (10). While some cancers depend on autophagy for survival, in some other model autophagy could suppress cancer development (9). The implication of autophagy in NB has also been reported by studies recently, and the results are also somewhat contradictory. Some studies reported tumor-suppressive role of autophagy for NB. For example, one study reported that inhibition of cyclooxygenase-2 (COX-2) promotes 1-methyl-4-phenyl 1,2,3,6 tetrahydropyridine (MPTP)-induced autophagic cell death in human NB cell line SH-SY5Y (17); another study revealed that Calcium/calmodulin-dependent protein kinase II (CAMK2) potentiates autophagic degradation of inhibitor of differentiation 1/2 (Id-1/2) and then induce cell differentiation of NB cells (14). However, there are also studies found tumor-protective role of autophagy for NB. For example, one study reported that activation of autophagy in human NB cell lines alleviates amyloid-β-induced apoptosis and neurotoxicity (15); another study reported that inhibition of unc-51 like autophagy kinase 1 (ULK1) significantly reduces cell growth and promotes cell apoptosis in NB cell lines (16); one study also reported that autophagy was also associated with chemoresistance of NB (13). It seems that autophagy also plays context-dependent roles in NB itself, maybe depending on activation of different autophagic pathways.

LncRNAs are known as RNA transcripts longer than 200 nucleotides with no or tiny protein-coding capacity, and are believed to play crucial roles in the development of various cancers including NB (20–25). The association between lncRNA and autophagy in NB is largely unknown. In this study, we focus on identifying autophagy-related lncRNAs that are associated with PFS of NB patients. The transcriptome profiles of one RNA-seq dataset and two microarray datasets with large number of NB tissue samples were utilized in this study to obtain a high confidence level. Out of 232 ARGs in the HADb, a total of 23 ARGs were found to be associated with PFS of NB patients, with 14 of them are risk factors and 9 are protective factors. The expression of a total of 752 lncRNAs were found to be correlated with the expression of the 23 PFS-related ARGs. However, only 11 of them were significantly associated with PFS of NB patients. After LASSO regression and multivariate Cox regression survival analyses, only four lncRNAs (AL356599.1, AC022075.1, AC020928.1 and LINC02076) were left as having the best prognostic value for PFS of NB patients, with two of them are risk factors (AC020928.1 and LINC02076) and another two (AL356599.1 and AC022075.1) are protective factors. The four lncRNAs were then incorporated into a lncRNA signature risk model for prediction of PFS of NB patients.

The four-lncRNA signature risk score successfully divided each of the three cohorts into two different risk groups, with patients in the low-risk group have relatively good survival outcome than patients in the high-risk group. We then combined the three cohorts together as one entire large cohort to perform further analyses in order to get more reliable results. Multivariate survival analyses performed in the combined cohort revealed that the prognostic role of this lncRNA signature for PFS is independent with other clinical risk factors (including age, MYCN amplification, INSS stage, and COG risk). The lncRNA signature also has good performance in the subgroup survival analyses stratified by different clinical risk factors. It significantly stratified both of COG low-risk patients and COG high-risk patients into two risk groups respectively, indicating that it could be used as a risk stratification factor along with the COG risk stratification system. Furthermore, we constructed a nomogram for predicting PFS of NB patients by incorporating the lncRNA signature risk score and the COG risk classification together. This nomogram revealed good consistency between predicted-PFS and actual-PFS. Moreover, the AUC of the nomogram was higher than the AUC of the COG risk alone, indicating that the lncRNA signature could help the COG risk classification system to predict PFS of NB patients with higher accuracy. These results suggest the use of this lncRNA signature as a risk stratification factor for NB.

The expression of these four lncRNAs are significantly corelated with the expression of autophagy-related genes DAPK1, ULK2, GNAI3, EIF2S1and MAP2K7 (with r ≥ 0.5). Data from this study revealed that high expression of DAPK1 and ULK2 are associated with good PFS of NB patients, while high expression of GNAI3, EIF2S1 and MAP2K7 are associated with bad PFS of NB patients (**Figure 1B**). For the two good ARGs, DAPK1, as a regulator of autophagy and apoptosis, is reported to function as a tumor-suppressor in various cancers (26), and has been reported to contribute to neuronal apoptosis due to ischemia reperfusion injury in mouse NB cell line N2a cells (27, 28); ULK2 has not been reported in NB, however, it has been reported to be required for proper projection of axons in the forebrain (29), indicating a tumor-suppressive role for NB. The function of DAPK1 and ULK2 reported previously are consistent with the results of our study, which suggest tumor-suppressive roles in NB. For the three bad ARGs, GNAI3 and EIF2S1 have not been reported to be implicated in NB previously, however, EIF2S1 has been reported to be involved with pathogenesis of neurodegenerative diseases as a target gene of transcript factor nuclear respiratory factor 1 (NRF1) (30); Downregulation of MAP2K7 (also known as MKK7) has been reported to be associated with decreased proliferation of NB cells (31). Of course, the function mechanisms of these ARGs in NB as well as their relationship with the lncRNAs need to be further investigated.

The exact functions of the four autophagy-related lncRNAs in cancers including NB are unknown. However, one of our previous study revealed that AL356599.1 (updated as FBXO30-DT, and previously known as LOC1005075557) and AC022075.1(updated as KLRK1-AS1, and previously known as LOC101928100) might be implicated in the process of spontaneous regression of NB, as both of them are differentially expressed between stage 4 and stage 4S NB samples and are correlated with the expression of NTRK1 (a well-known factor involved in spontaneous regression of NB) (32–34). This result indicates that autophagy might be also participated in the spontaneous regression of NB, since these two lncRNAs are related to autophagic genes. As for AC020928.1 (updated as LOC728485) and LINC02076, no literature has reported their specific roles in cancers including NB. The exact roles of these four lncRNAs in the development of NB and the underling mechanisms as well as their relationship with autophagy need to be clarified by further studies.

It is also very interesting that the GESA analyses revealed no autophagy-related gene set enriched in the high-risk group, while multiple autophagy-related gene sets (GO selective autophagy, GO positive regulation of autophagy, GO positive regulation of macroautophagy, GO autophagosome organization, GO negative regulation of autophagy, GO regulation of autophagy, GO positive regulation of autophagy of mitochondrion, and KEGG regulation of autophagy) were significantly enriched in the low-risk group. These results suggest that autophagy tend to play tumor-suppressive roles in NB, which is consistent with previous studies revealing tumor-suppressive role of autophagy in NB (14, 17). However, this result is somewhat contrary to some of the previous studies revealing tumor-protective roles of autophagy in NB (15, 16). We presume that autophagy plays context-dependent roles in NB itself, and different autophagic genes or pathways might play different roles. Undoubtedly, further investigations are needed to clarify how different autophagic pathways affect the progression of NB, thus providing guidance for autophagy-related therapeutic strategies in NB patients.

## Conclusion

In conclusion, we find that, in the tumor tissue level, autophagy is associated with the progression of NB. An autophagy-related four-lncRNA prognostic signature was built and performed well in predicting PFS of NB patients. The four-lncRNA signature is independent with other clinical risk factors and also helps the COG risk classification to better predict the PFS of NB patients with more accuracy. Our study provides potentially promising ways to obtain a prognosis for NB patients and show that autophagy associated therapies may be a potential treatment for NB.

## Data availability statement

The original contributions presented in the study are included in the article/**Supplementary Material**. Further inquiries can be directed to the corresponding author.

## Author contributions

JF designed the study. JW, KC, and XM performed the data analysis. JW and XM wrote the original manuscript. JF revised the manuscript and provided funding acquisition. All authors contributed to the article and approved the submitted version.

## Funding

This research was supported by the National Natural Science Foundation of China (grant number 81873541).

## Conflict of interest

The authors declare that the research was conducted in the absence of any commercial or financial relationships that could be construed as a potential conflict of interest.

## Publisher’s note

All claims expressed in this article are solely those of the authors and do not necessarily represent those of their affiliated organizations, or those of the publisher, the editors and the reviewers. Any product that may be evaluated in this article, or claim that may be made by its manufacturer, is not guaranteed or endorsed by the publisher.

## Abbreviations

LncRNA, long non-coding RNA; NB, neuroblastoma; PFS, progression-free survival; ARG, autophagy-related gene; LASSO, least absolute shrinkage and selection operator; ROC, receiver operating characteristic; AUC, area under curve; GSEA, Gene Set Enrichment Analysis; COG, Children’s Oncology Group; INSS, International Neuroblastoma Staging System; OS, overall survival; GEO, Gene Expression Omnibus; HADb, Human Autophagy Database; FDR, false discovery rate; COX-2, cyclooxygenase-2; MPTP, 1-methyl-4-244 phenyl 1,2,3,6 tetrahydropyridine; CAMK2, Calcium/calmodulin-dependent protein kinase II; Id-1/2, inhibitor of differentiation 1/2; ULK1, unc-51-like autophagy kinase 1.

## References

[1] Gurney JG Ross JA Wall DA Bleyer WA Severson RK Robison LL. Infant cancer in the U.S.: Histology-specific incidence and trend 1973 to 1992. J Pediatr Hematol Oncol (1997) 19(5):428–32. doi: 10.1097/00043426-199709000-00004 9329464

[2] London WB Castleberry RP Matthay KK Look AT Seeger RC Shimada H. Evidence for an age cutoff greater than 365 days for neuroblastoma risk group stratification in the children's oncology group. J Clin Oncol (2005) 23(27):6459–65. doi: 10.1200/jco.2005.05.571 16116153

[3] Maris JM. Recent advances in neuroblastoma. N Engl J Med (2010) 362(23):2202–11. doi: 10.1056/NEJMra0804577 PMC3306838 20558371

[4] Strother DR London WB Schmidt ML Brodeur GM Shimada H Thorner P. Outcome after surgery alone or with restricted use of chemotherapy for patients with low-risk neuroblastoma: Results of children's oncology group study P9641. J Clin Oncol (2012) 30(15):1842–8. doi: 10.1200/JCO.2011.37.9990 PMC3383182 22529259

[5] Baker DL Schmidt ML Cohn SL Maris JM London WB Buxton A. Outcome after reduced chemotherapy for intermediate-risk neuroblastoma. New Engl J Med (2010) 363(14):1313–23. doi: 10.1056/NEJMoa1001527 PMC2993160 20879880

[6] Mertens AC Yasui Y Neglia JP Potter JD Nesbit ME Ruccione K. Late mortality experience in five-year survivors of childhood and adolescent cancer: the childhood cancer survivor study. J Clin Oncol (2001) 19(13):3163–72. doi: 10.1200/JCO.2001.19.13.3163 11432882

[7] Cotterill SJ Pearson AD Pritchard J Kohler JA Foot AB. Late relapse and prognosis for neuroblastoma patients surviving 5 years or more: A report from the European neuroblastoma study group "Survey". Med Pediatr Oncol (2001) 36(1):235–8. doi: 10.1002/1096-911X(20010101)36:1<235::AID-MPO1057>3.0.CO;2-N 11464893

[8] Chen P Cescon M Bonaldo P. Autophagy-mediated regulation of macrophages and its applications for cancer. Autophagy (2014) 10(2):192–200. doi: 10.4161/auto.26927 24300480 PMC5396097

[9] White E Mehnert JM Chan CS. Autophagy, metabolism, and cancer. Clin Cancer Res (2015) 21(22):5037–46. doi: 10.1158/1078-0432.Ccr-15-0490 PMC4646728 26567363

[10] Levy JMM Towers CG Thorburn A. Targeting autophagy in cancer. nature reviews. Cancer (2017) 17(9):528–42. doi: 10.1038/nrc.2017.53 PMC5975367 28751651

[11] Levy JMM Thorburn A. Targeting autophagy during cancer therapy to improve clinical outcomes. Pharmacol Ther (2011) 131(1):130–41. doi: 10.1016/j.pharmthera.2011.03.009 PMC3539744 21440002

[12] Towers CG Thorburn A. Therapeutic targeting of autophagy. EBioMedicine (2016) 14:15–23. doi: 10.1016/j.ebiom.2016.10.034 28029600 PMC5161418

[13] Belounis A Nyalendo C Le Gall R Imbriglio TV Mahma M Teira P. Autophagy is associated with chemoresistance in neuroblastoma. BMC Cancer (2016) 16(1):891. doi: 10.1186/s12885-016-2906-9 27846885 PMC5109645

[14] Li X Wu XQ Deng R Li DD Tang J Chen WD. CaMKII-mediated beclin 1 phosphorylation regulates autophagy that promotes degradation of id and neuroblastoma cell differentiation. Nat Commun (2017) 8(1):1159. doi: 10.1038/s41467-017-01272-2 29079782 PMC5660092

[15] Singh AK Bissoyi A Kashyap MP Patra PK Rizvi SI. Autophagy activation alleviates amyloid-β-Induced oxidative stress, apoptosis and neurotoxicity in human neuroblastoma SH-SY5Y cells. Neurotoxicity Res (2017) 32(3):351–61. doi: 10.1007/s12640-017-9746-5 28484969

[16] Dower CM Bhat N Gebru MT Chen L Wills CA Miller BA. Targeted inhibition of ULK1 promotes apoptosis and suppresses tumor growth and metastasis in neuroblastoma. Mol Cancer Ther (2018) 17(11):2365–76. doi: 10.1158/1535-7163.Mct-18-0176 PMC6215526 30166400

[17] Niranjan R Mishra KP Thakur AK. Inhibition of cyclooxygenase-2 (COX-2) initiates autophagy and potentiates MPTP-induced autophagic cell death of human neuroblastoma cells, SH-SY5Y: An inside in the pathology of parkinson's disease. Mol Neurobiol (2018) 55(10):8038–50. doi: 10.1007/s12035-018-0950-y 29498006

[18] Sha Z Schnell HM Ruoff K Goldberg A. Rapid induction of p62 and GABARAPL1 upon proteasome inhibition promotes survival before autophagy activation. J Cell Biol (2018) 217(5):1757–76. doi: 10.1083/jcb.201708168 PMC5940303 29535191

[19] Tibshirani R. The lasso method for variable selection in the cox model. Stat Med (1997) 16(4):385–95. doi: 10.1002/(sici)1097-0258(19970228)16:4<385 9044528

[20] Ma L Bajic VB Zhang Z. On the classification of long non-coding RNAs. RNA Biol (2013) 10(6):925–33. doi: 10.4161/rna.24604 PMC4111732 23696037

[21] Russell MR Penikis A Oldridge DA Alvarez-Dominguez JR McDaniel L Diamond M. CASC15-s is a tumor suppressor lncRNA at the 6p22 neuroblastoma susceptibility locus. Cancer Res (2015) 75(15):3155–66. doi: 10.1158/0008-5472.Can-14-3613 PMC4526355 26100672

[22] Bhan A Soleimani M Mandal SS. Long noncoding RNA and cancer: A new paradigm. Cancer Res (2017) 77(15):3965–81. doi: 10.1158/0008-5472.Can-16-2634 PMC8330958 28701486

[23] Tang Y Cheung BB Atmadibrata B Marshall GM Dinger ME Liu PY. The regulatory role of long noncoding RNAs in cancer. Cancer Lett (2017) 391:12–9. doi: 10.1016/j.canlet.2017.01.010 28111137

[24] Li D Wang X Mei H Fang E Ye L Song H. Long noncoding RNA pancEts-1 promotes neuroblastoma progression through hnRNPK-mediated beta-catenin stabilization. Cancer Res (2018) 78(5):1169–83. doi: 10.1158/0008-5472.Can-17-2295 29311158

[25] Liu PY Tee AE Milazzo G Hannan KM Maag J Mondal S. The long noncoding RNA lncNB1 promotes tumorigenesis by interacting with ribosomal protein RPL35. Nat Commun (2019) 10(1):5026. doi: 10.1038/s41467-019-12971-3 31690716 PMC6831662

[26] Singh P Ravanan P Talwar P. Death associated protein kinase 1 (DAPK1): A regulator of apoptosis and autophagy. Front Mol Neurosci (2016) 9:46. doi: 10.3389/fnmol.2016.00046 27445685 PMC4917528

[27] Xiong W Wu Y Xian W Song L Hu L Pan S. DAPK1-ERK signal mediates oxygen glucose deprivation reperfusion induced apoptosis in mouse N2a cells. J Neurol Sci (2018) 387:210–9. doi: 10.1016/j.jns.2018.01.003 29571866

[28] Wei R Zhang L Hu W Wu J Zhang W. Long non-coding RNA AK038897 aggravates cerebral ischemia/reperfusion injury *via* acting as a ceRNA for miR-26a-5p to target DAPK1. Exp Neurol (2019) 314:100–10. doi: 10.1016/j.expneurol.2019.01.009 30703362

[29] Wang B Iyengar R Li-Harms X Joo JH Wright C Lavado A. The autophagy-inducing kinases, ULK1 and ULK2, regulate axon guidance in the developing mouse forebrain *via* a noncanonical pathway. Autophagy (2018) 14(5):796–811. doi: 10.1080/15548627.2017.1386820 29099309 PMC6070005

[30] Satoh J-I Kawana N Yamamoto Y. Pathway analysis of ChIP-Seq-Based NRF1 target genes suggests a logical hypothesis of their involvement in the pathogenesis of neurodegenerative diseases. Gene Regul Syst Biol (2013) 7:139–52. doi: 10.4137/GRSB.S13204 PMC3825669 24250222

[31] He W Wu Y Tang X Xia Y He G Min Z. HDAC inhibitors suppress c-Jun/Fra-1-mediated proliferation through transcriptionally downregulating MKK7 and Raf1 in neuroblastoma cells. Oncotarget (2016) 7(6):6727–47. doi: 10.18632/oncotarget.6797 PMC4872745 26734995

[32] Brodeur GM Minturn JE Ho R Simpson AM Iyer R Varela CR. Trk receptor expression and inhibition in neuroblastomas. Clin Cancer Res (2009) 15(10):3244–50. doi: 10.1158/1078-0432.CCR-08-1815 PMC4238907 19417027

[33] Thiele CJ Li Z McKee AE. On trk–the TrkB signal transduction pathway is an increasingly important target in cancer biology. Clin Cancer Res (2009) 15(19):5962–7. doi: 10.1158/1078-0432.CCR-08-0651 PMC2756331 19755385

[34] Meng X Fang E Zhao X Feng J. Identification of prognostic long noncoding RNAs associated with spontaneous regression of neuroblastoma. Cancer Med (2020) 9(11):3800–15. doi: 10.1002/cam4.3022 PMC7286466 32216054

