# Supplementary material for: An autophagy-related four-lncRNA signature helps to predict progression-free survival of neuroblastoma patients
Source: Front Oncol. 2022 Dec 1;12:1014845. doi: 10.3389/fonc.2022.1014845 (PMC9753905; doi:10.3389/fonc.2022.1014845)
Supplement: Supplementary file 1 [file Table_1.docx]

**Table S1**

**Table S1.** The clinical characteristics of the three cohorts.

|  | **No. of cohort 1 (%)** | **No. of cohort 2 (%)** | **No. of cohort 3 (%)** |
| --- | --- | --- | --- |
| **Age** |  |  |  |
| < 18 months | 29 (19.0%) | 300 (60.2%) | 103 (46.2%) |
| ≥ 18 months | 124 (81.0%) | 198 (39.8%) | 120 (53.8%) |
| **Gender** |  |  |  |
| Male | 89 (58.2%) | 287 (57.6%) | - |
| Female | 64 (41.8%) | 211 (42.4%) | - |
| **MYCN status** |  |  |  |
| Not-amplified | 121 (79.1%) | 401 (80.5%) | 176 (78.9%) |
| Amplified | 31 (20.3%) | 92 (18.5%) | 46 (20.6%) |
| **COG Risk** |  |  |  |
| Low | 13 (8.5%) | 322 (64.7%) | - |
| Intermediate | 14(9.1%) | 0 | - |
| High | 126 (82.4%) | 176 (35.3%) | - |
| **INSS Stage** |  |  |  |
| 1 | 0 | 121 (24.3%) | 29 (13.0%) |
| 2 | 1 (0.7%) | 78 (15.7%) | 39 (17.5%) |
| 3 | 6 (3.9%) | 63 (12.7%) | 36 (16.1%) |
| 4 | 125 (81.7%) | 183 (36.7%) | 89 (39.9%) |
| 4S | 21 (13.7%) | 53 (10.6%) | 30 (13.5%) |
| **Survival status** |  |  |  |
| Dead | 77 (50.3%) | 105 (21.1%) | 42 (18.8%) |
| Alive | 76 (49.7%) | 393 (78.9%) | 181 (81.2%) |
| **Progression status** |  |  |  |
| Yes | 96(62.7%) | 183(36.7%) | 89(39.9%) |
| No | 57(37.3%) | 315(63.3%) | 134(60.1%) |
