# Supplementary material for: An autophagy-related four-lncRNA signature helps to predict progression-free survival of neuroblastoma patients
Source: Front Oncol. 2022 Dec 1;12:1014845. doi: 10.3389/fonc.2022.1014845 (PMC9753905; doi:10.3389/fonc.2022.1014845)
Supplement: Supplementary file 2 [file DataSheet_1.docx]

**Figure S1**


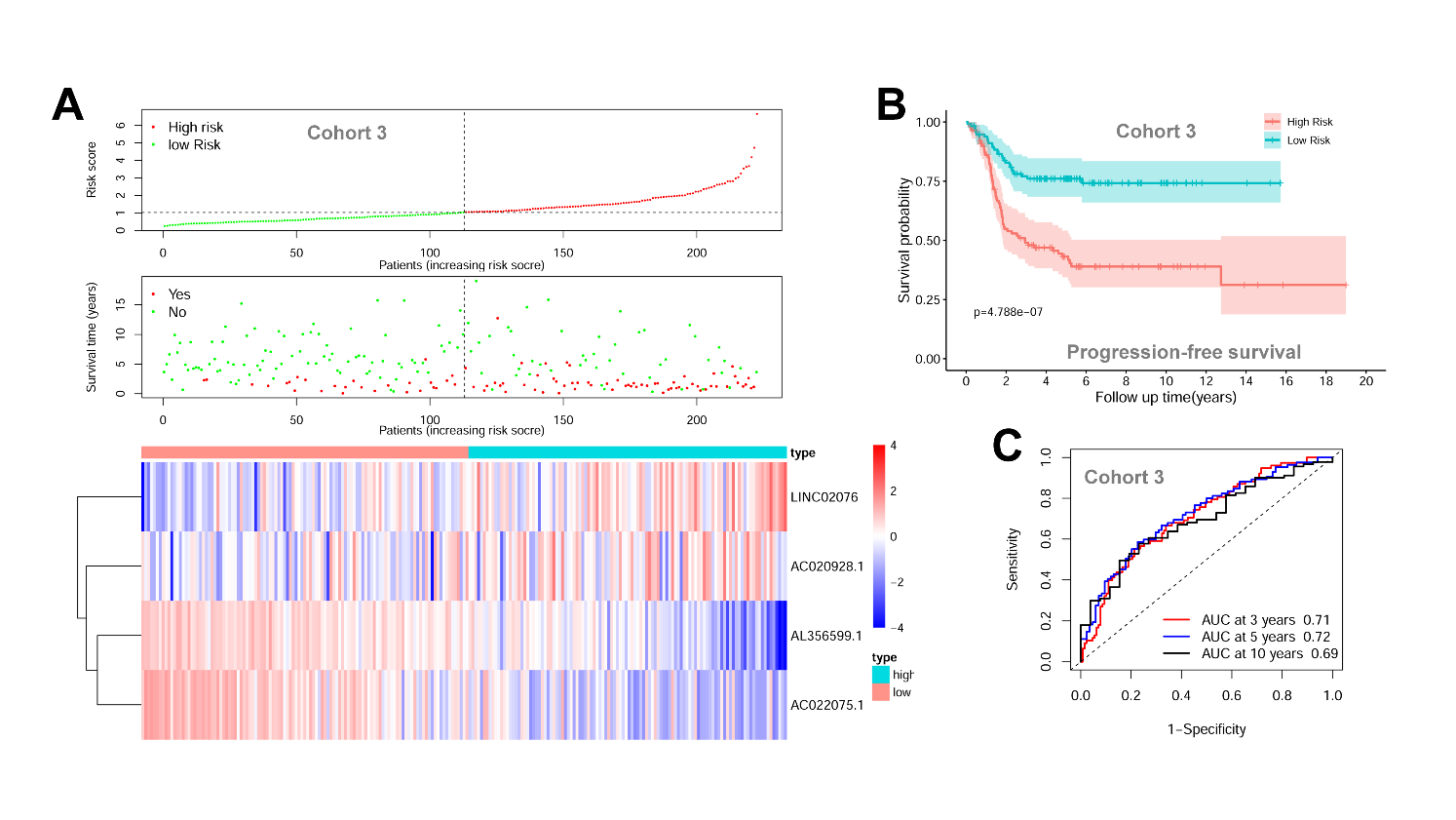


**Figure S1.** The autophagy-related four-lncRNA signature risk score in cohort 3. (A) The distribution of risk scores, survival status of each patient, and heatmap of lncRNAs expression pattern in cohort 1. (B) Kaplan-Meier survival curve for PFS of patients in the low-risk group and high-risk group for cohort 3. (C) Time-dependent ROC curves of the lncRNA signature in cohort 3.

**Figure S2**


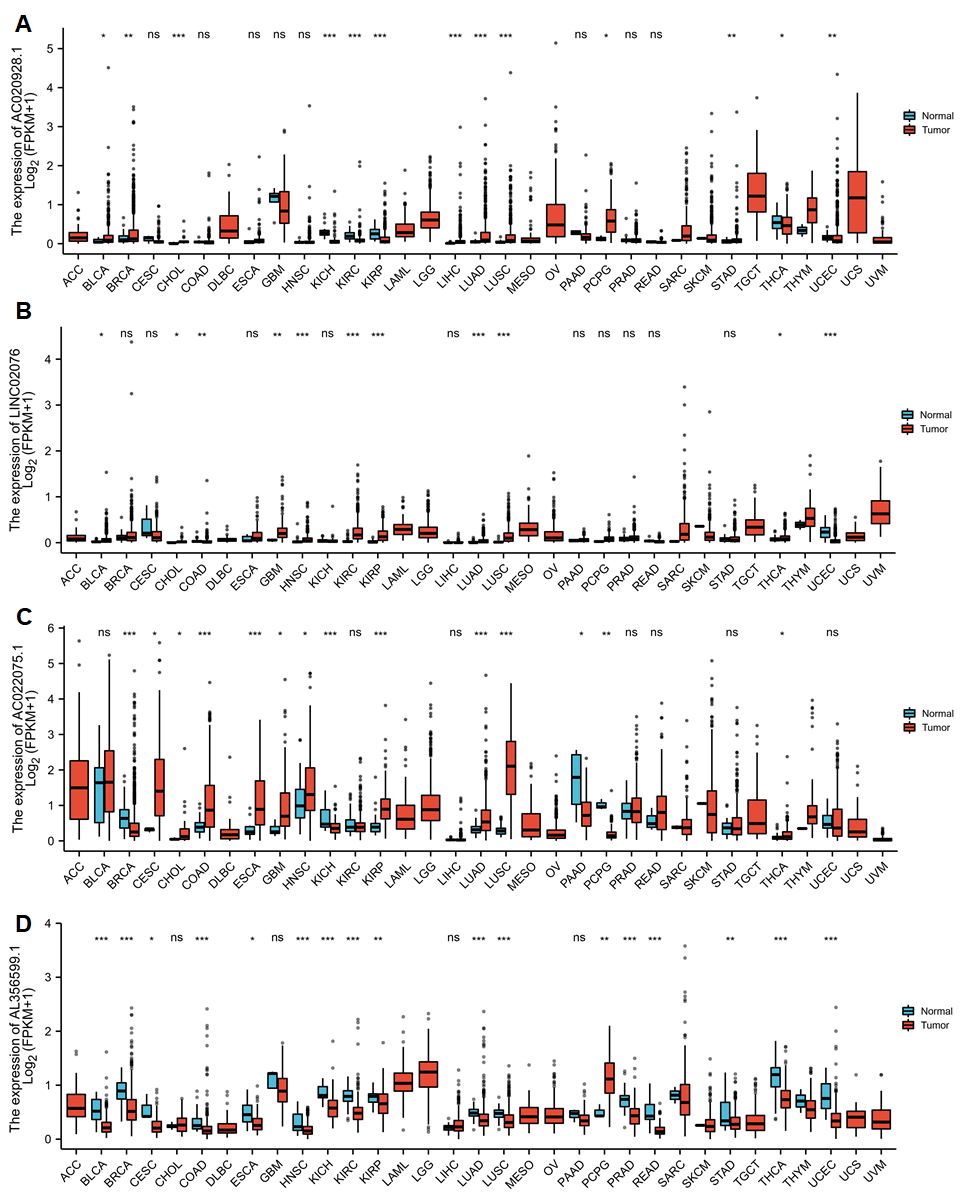


**Figure S2.** The differential analysis of the autophagy-related four-lncRNA (A) AC020928.1, (B) LINC02076, (C) AC022075.1, and (D) AL356599.1 in Pan-cancer. ^ns^ *p*>0.05; * *p*<0.05; ** *p*<0.01; *** *p*<0.001; **** *p*<0.0001.

**Figure S3**


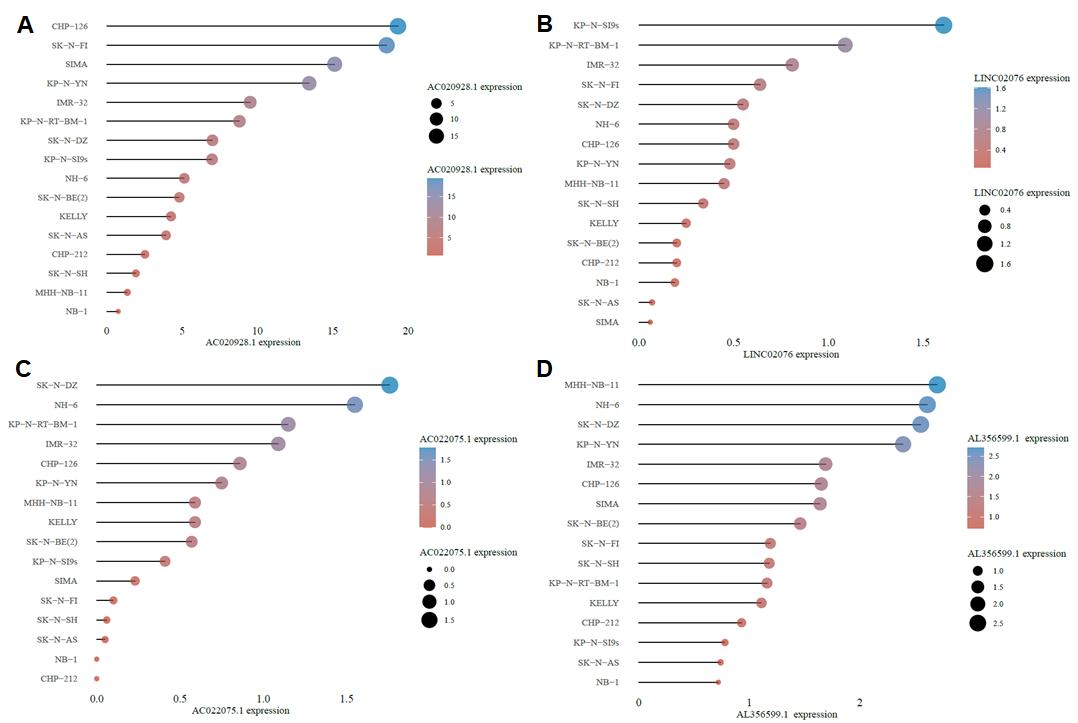


**Figure S3.** The expression of these lncRNAs in 16 types of NB cell lines: (A) AC020928.1, (B) LINC02076, (C) AC022075.1, and (D) AL356599.1.
